# Study on shock vibration analysis and foundation reinforcement of large ball mill

**DOI:** 10.1038/s41598-022-26194-y

**Published:** 2023-01-05

**Authors:** Qu Tie, Tang Biliang, Bian Qiang, Zhang Xiangyun, Chen Ming, Zhao Chunjiang

**Affiliations:** 1 State Key Laboratory of Mining Heavy Equipment, CITIC Heavy Industry Machinery Co., Ltd., Luoyang, 471000 China; 2 School of Mechanical Engineering, Taiyuan University of Science and Technology, Taiyuan, 030024 China

**Keywords:** Mechanical engineering, Civil engineering

## Abstract

The vibration of the ball mill foundation will seriously affect the operation stability of the ball mill. According to the connection relationship between the ball mill and the foundation, the mill-foundation transient shock model is established. The effects of shock force, concrete grade and shock angle on the transient response of the foundation were studied. And based on the problem of the mill foundation, the reinforcement scheme of the foundation was put forward. The following conclusions are drawn: with the increase of shock force and shock angle, the displacement of foundation joints increases, and the maximum stress also increases; concrete grade has little effect on foundation stability; after the foundation reinforcement scheme is used, the vibration caused by the shock of the ball mill is effectively reduced, and the stability of the foundation is increased. Finally, the correctness of the model and the scheme is verified through the comparison with the test data. The calculation results provide a reference for the improvement of the ball mill foundation.

## Introduction

The ball mill is the key equipment for grinding the minerals after the ore is crushed. With the continuous development of the industrial level, the development of ball mills is also moving towards the trend of large-scale^1–4^. Due to the large shock force generated during the operation of the large ball mill, the foundation of the ball mill will vibrate^5,6^. The vibration will lead to uneven meshing of large and small gears, resulting in additional force, which seriously affects the normal operation of the ball mill as a whole. Therefore, it is very important to analyze the shock and vibration of the ball mill foundation^7^.

Many scholars have done in-depth research on the foundation. In the 1860s, Manoharan^8^ gave a finite element viscoelastic-plastic solution to the general bearing capacity problem of strip and circular surface foundations. Nulu et al.^9^ used ANSYS software to study the ground response of equipment startup and shutdown conditions (transition phase). Li Xin et al.^10^ used ABAQUS to simulate the dynamic response of subgrade in karst area under the action of train dynamic load. Through experimental research, Guangya Ding et al.^11^ analyzed the performance of soil bag reinforcement for roadbed from three aspects: vibration acceleration, earth pressure and vertical deformation. Shujun Yan et al.^12^ established a finite element model of the dynamic response of the subgrade structure under the action of train loads, and analyzed the vibration and dynamic stress of the high embankment and heavy-duty railway subgrade. Li Shaoyi et al.^13^ used 2.5-dimensional finite element method to study the effect of high train speed on the vibration of saturated roadbed. Qian Jiangu et al.^14^ conducted a theoretical study on the dynamic response of saturated foundation under high-speed moving loads. Jiang Hongguang et al.^15^ established a finite element analysis model considering the uneven settlement of the subgrade, and studied the effect of uneven settlement on the vibration propagation of the foundation under the condition of increasing the speed of the train. Kang Yanwu^16^ established the finite element model of the mill-base system, and carried out modal analysis and transient analysis respectively.

For the transient impact of the ball mill, the ball mill foundation of CITIC Heavy Industry was taken as the research object, and a finite element model of the ball mill foundation was established based on the ABAQUS software. The effects of different shock force, concrete grades and shock angles on the vibration characteristics of the foundation are studied. Then, based on the existing problems of the mill foundation, a reinforcement scheme is proposed, and the simulation results and the foundation reinforcement scheme are verified by the experimental data.

### Foundational modal vibration theory

The mode of the system is an inherent characteristic, and the modes under different conditions correspond to specific frequencies and mode shapes. Through the modal analysis of the system, the interaction between the system structure and other systems can be predicted, so that the generation of resonance can be suppressed by changing the design of the structure^16^.

For the modal calculation of a system, it is first necessary to divide the whole system into s subsystems, and the modal matrix $\left[ {{\varvec{\Phi}}} \right]$ of each subsystem is:

1 $$ \left[ {{\varvec{\Phi}}} \right]{\mathbf{ = }}\left[ {\left[ {{{\varvec{\Phi}}}^{{\mathbf{F}}} } \right] \vdots \left[ {{{\varvec{\Phi}}}^{{\mathbf{B}}} } \right]} \right] $$

In the formula, $\left[ {{{\varvec{\Phi}}}^{{\mathbf{F}}} } \right]$ is the natural modes of the completely fixed interface in the system, that is, the main mode matrix; $\left[ {{{\varvec{\Phi}}}^{{\mathbf{B}}} } \right]$ is the constraint modal matrix composed of the static displacement distribution generated by releasing each boundary degree of freedom(DOF) in turn to obtain unit displacement.

When the restrained modes are calculated, a series of motion equations of the subsystem with unit forced displacement at the boundary DOF are:

2 $$ \left[ {\frac{{{\text{k}}_{{{\text{ii}}}} }}{{{\text{k}}_{{{\text{ji}}}} }} \vdots \frac{{{\text{k}}_{{{\text{ij}}}} }}{{{\text{k}}_{{{\text{jj}}}} }}} \right] \bullet \left[ {\frac{{{\text{u}}_{{\text{i}}} }}{{{\text{u}}_{{\text{j}}} }}} \right] = \left[ {\frac{{0}}{{\text{F}}}} \right] $$

where ${\text{u}}_{{\text{i}}}$ is the internal coordinate, ${\text{u}}_{{\text{j}}}$ is the interface coordinate, ${\text{k}}$ is the stiffness of the system, and F is the external force.

The boundary DOF generate unit displacements in turn, that is, ${\text{u}}_{{\text{j}}}$ is the unit matrix. Expand formula (2) from the first row to get:

3 $$ \left[ {{\text{k}}_{{{\text{ii}}}} } \right] \bullet \left[ {{\text{u}}_{{\text{i}}} } \right] + \left[ {{\text{k}}_{{{\text{ij}}}} } \right] \bullet \left[ {\text{E}} \right] = \left[ 0 \right] $$

where E is the identity matrix, which can be simplified to get:

4 $$ \left[ {{\text{u}}_{{\text{i}}} } \right] = - \left[ {{\text{k}}_{{{\text{ii}}}} } \right]^{ - 1} \bullet \left[ {{\text{k}}_{{{\text{ij}}}} } \right] $$

The constraint modal matrix $\left[ {{{\varvec{\Phi}}}^{{\mathbf{B}}} } \right]$ can be obtained as:

5 $$ \left[ {{{\varvec{\Phi}}}^{{\mathbf{B}}} } \right] = \left[ {\frac{{{\text{u}}_{{\text{i}}} }}{{\text{E}}}} \right] = \left[ {\frac{{ - \left[ {{\text{k}}_{{{\text{ii}}}} } \right]^{ - 1} \bullet \left[ {{\text{k}}_{{{\text{ij}}}} } \right]}}{{\text{E}}}} \right] $$

It can be seen from formula (5) that the column number of $\left[ {{{\varvec{\Phi}}}^{{\mathbf{B}}} } \right]$ is the number of matrix boundary DOF.

6 $$ \left[ {{{\varvec{\Phi}}}_{{}}^{{\mathbf{F}}} } \right] = \left[ {\frac{{\left[ {{{\varvec{\Phi}}}_{{\text{n}}}^{{\mathbf{F}}} } \right]}}{0}} \right] $$

7 $$ \left[ {{\varvec{\Phi}}} \right]{\mathbf{ = }}\left[ {\left[ {{{\varvec{\Phi}}}^{{\mathbf{F}}} } \right] \vdots \left[ {{{\varvec{\Phi}}}^{{\mathbf{B}}} } \right]} \right] = \left[ {\frac{{\left[ {{{\varvec{\Phi}}}_{{\text{n}}}^{{\mathbf{F}}} } \right]}}{0} \vdots \frac{{{\text{u}}_{{\text{i}}} }}{{\text{E}}}} \right] $$

The transformed equation of motion is:

8 $$ {\text{m}} \bullet {\ddot{\text{p}}} + {\text{k}} \bullet {\text{p}} = 0 $$

The modal matrix $\left[ {{\varvec{\Phi}}} \right]$ is used to perform the first coordinate transformation of the subsystem mass moment $\left[ {{\overline{\text{m}}}} \right]$ and stiffness matrix $\left[ {{\overline{\text{k}}}} \right]$ to obtain:

9 $$ \left[ {\text{k}} \right] = \left[ {{\varvec{\Phi}}} \right]^{{\text{T}}} \left[ {{\overline{\text{k}}}} \right]\left[ {{\varvec{\Phi}}} \right],\;\left[ {\text{m}} \right] = \left[ {{\varvec{\Phi}}} \right]^{{\text{T}}} \left[ {{\overline{\text{m}}}} \right]\left[ {{\varvec{\Phi}}} \right] $$

Then the interconnection between subsystems can be realized through the second coordinate. When there is a common boundary surface between the r subsystem and the q subsystem, the displacement vectors are:

10 $$ \left[ {{\text{p}}_{{\text{r}}} } \right] = \left[ {\frac{{{\text{p}}_{{{\text{rF}}}} }}{{{\text{p}}_{{{\text{rB}}}} }}} \right],\;\left[ {{\text{p}}_{{\text{q}}} } \right] = \left[ {\frac{{{\text{p}}_{{{\text{qF}}}} }}{{{\text{p}}_{{{\text{qB}}}} }}} \right] $$

The subscript B denotes the constrained modal generalized displacement on the common boundary surface. Since the displacement at the intersection point of the boundary surface must be equal, the generalized displacement of the constraint mode corresponding to the displacement is equal:

11 $$ \left[ {{\text{p}}_{{{\text{rB}}}} } \right] = \left[ {{\text{p}}_{{{\text{qB}}}} } \right] $$

12 $$ \left[ {\text{p}} \right] = \left[ {\frac{{{\text{p}}_{{\text{r}}} }}{{{\text{p}}_{{\text{q}}} }}} \right] = \left[ {\begin{array}{*{20}c} {{\text{p}}_{{{\text{rF}}}} } \\ {{\text{p}}_{{{\text{rB}}}} } \\ {{\text{p}}_{{{\text{qF}}}} } \\ {{\text{p}}_{{{\text{qB}}}} } \\ \end{array} } \right] = \left[ {\begin{array}{*{20}c} {\left[ {\text{E}} \right]} & 0 & 0 \\ 0 & {\left[ {\text{E}} \right]} & 0 \\ 0 & {\left[ {\text{E}} \right]} & 0 \\ 0 & 0 & {\left[ {\text{E}} \right]} \\ \end{array} } \right] \bullet \left[ {\begin{array}{*{20}c} {{\text{p}}_{{{\text{rF}}}} } \\ {{\text{p}}_{{{\text{rB}}}} } \\ {{\text{p}}_{{{\text{qF}}}} } \\ \end{array} } \right] = \left[ \beta \right] \bullet \left[ {{\text{p}}^{{\text{c}}} } \right] $$

Substitute Eq. (12) into Eq. (8), that is, use $\left[ {{\text{p}}^{{\text{c}}} } \right]$ to perform the second coordinate transformation instead of the original system $\left[ {\text{p}} \right]$, and then left-multiply $\left[ \beta \right]^{{\text{T}}}$ to get:

13 $$ \left[ {\text{K}} \right] = \left[ \beta \right]^{{\text{T}}} \left[ {\text{k}} \right]\left[ \beta \right],\;\left[ {\text{M}} \right] = \left[ \beta \right]^{{\text{T}}} \left[ {\text{m}} \right]\left[ \beta \right] $$

Finally, the generalized eigenvalue equation is obtained:

14 $$ \left[ K \right] \bullet \left[ x \right] = \omega^{2} \left[ M \right]\left[ x \right] $$

By solving Eq. (14), the constrained modal characteristics of the system can be obtained.

### The establishment of the finite element model

The finite element method can be used to analyze the modal and modal dynamics of complex structures with high calculation accuracy. Therefore, the finite element software is used to complete the modal analysis of the ball mill foundation. Taking the φ7.32 × 12.5 m ball mill foundation as the research object, the foundational length is 33 m, the width is 16 m, and the height is 6 m. Models such as foundations, cylinders, bearing seats are established in SolidWorks software, and then imported into ABAQUS software. The material of each component is set in the property unit, and the material parameters are shown in Table 1. In order to fit the actual situation, the cylinder body, foundation and bearing bush are set as elastic bodies, and the soil base is set as viscoelastic body.

**Table 1:** Basic parameters of ball mill foundation model.

Part	Material	Density kg/m^3^	Elastic modulus E/MPa	Poisson's ratio
Cylinder	ASTM A36 /Q235B	7800	196,000	0.28
Soil foundation	Tight sand	1800	100	0.3
Bush	Copper alloy	8800	128,000	0.33
Gear	ZG40CrNi2Mo	7850	210,000	0.31
Concrete foundation	C35	2420	31,500	0.2

Modal analysis and modal dynamics are both linear perturbation calculations. The modal dynamics calculation is based on the modal analysis to obtain the dynamic response of the system under the action of the time-varying load. In the step element, the modal analysis step is first established, and the Lanczos solver is used to solve the problem. The number of eigenvalues is selected by the numerical method and input 50, that is, the first 50 vibration modes are extracted. Then, a modal dynamics analysis step is established on the basis of the modal analysis step.

**Figure 1:**
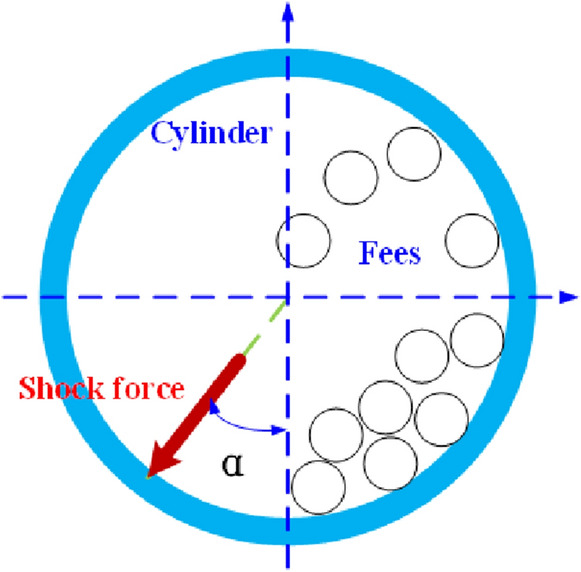
Schematic diagram of shock angle.

In order to simulate the actual working conditions, binding constraints are added between the cylinder and the bearing pad, the bearing pad and the bearing seat, and the bearing seat and the concrete foundation. The motor and the bearing seat placement surface are respectively coupled to one point, and a force is applied to the coupling point. The model is applied with a gravity of 9.8 m/s^2^, and a shock force is applied to the material blanking part inside the cylinder. The shock force action time is set to 0.01 s, and the value of shock angle is according to the actual impact of the blanking on the cylinder. The shock angle is defined as the angle between the shock force direction and the vertical direction, which is represented by α. Figure 1 is a schematic diagram of the shock angle.

**Figure 2:**
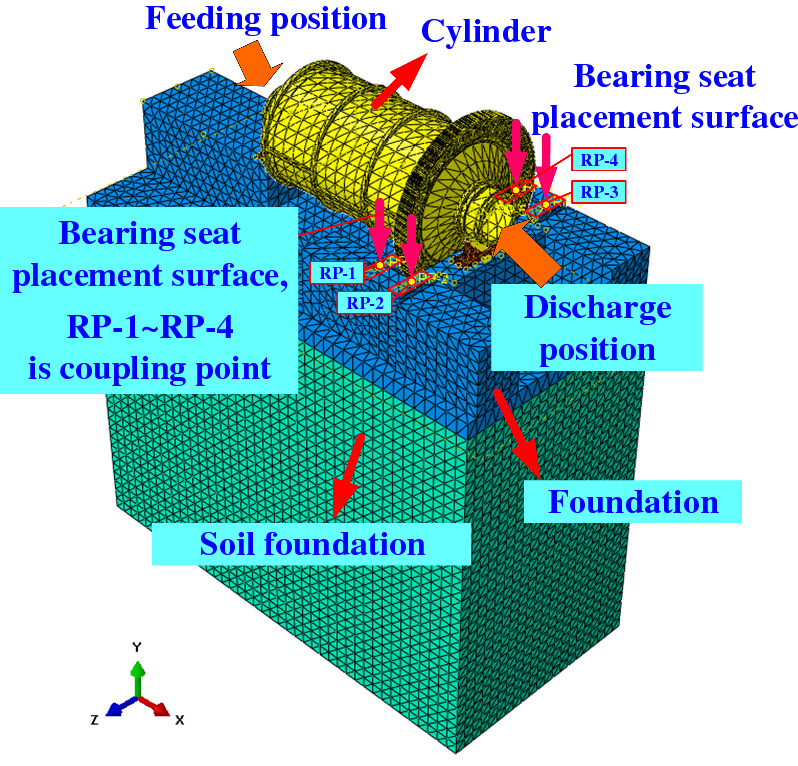
Finite element model of ball mill foundation.

Constrain the 6 DOF of the soil foundation in the boundary conditions. Due to the large size of the model and the complex structure, considering the accuracy and efficiency of the calculation, the element type of each component of the bearing adopts the tetrahedral element, and the type of the tetrahedral element is assigned as C3D10. The finite element model is shown in Fig. 2.

## Results and analysis

**Figure 3:**
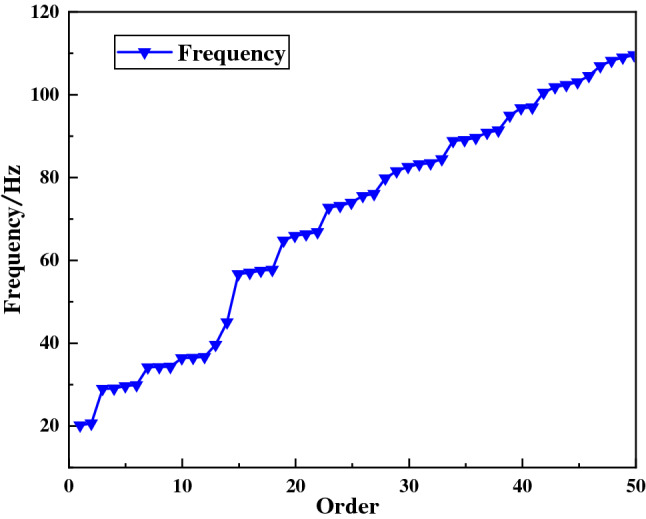
The natural frequency curve of the ball mill.

**Figure 4:**
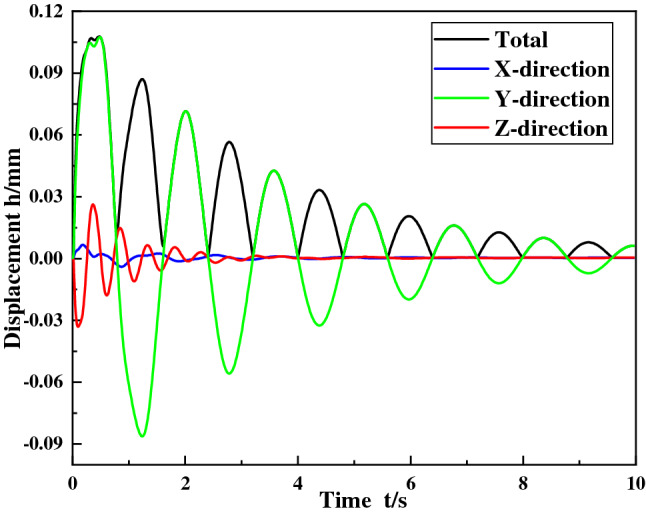
Displacement curve of foundation node at discharge position.

When the shock angle is 30°, the concrete grade is C35, and the shock force is 12,500 kN, the shock vibration response of the ball mill foundation is obtained. The first 50 order natural frequencies of the ball mill and the displacement curves of the foundational nodes at the discharge position of the cylinder are shown in Figs. 3 and 4. It can be seen from figures that the natural frequency of the ball mill system increases with the increase of the order, and the frequency range of the first 50 orders is 19–110 Hz. The total displacement of the foundation node at the discharge end is attenuated periodically, the maximum displacement is 0.107 mm, and the displacement is the largest in the Y direction.

### Influence of force

**Figure 5:**
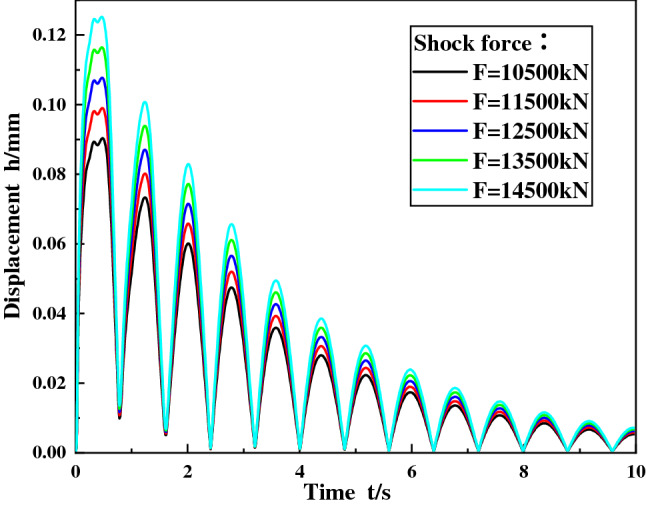
Displacement curve of foundation node under different shock force.

**Figure 6:**
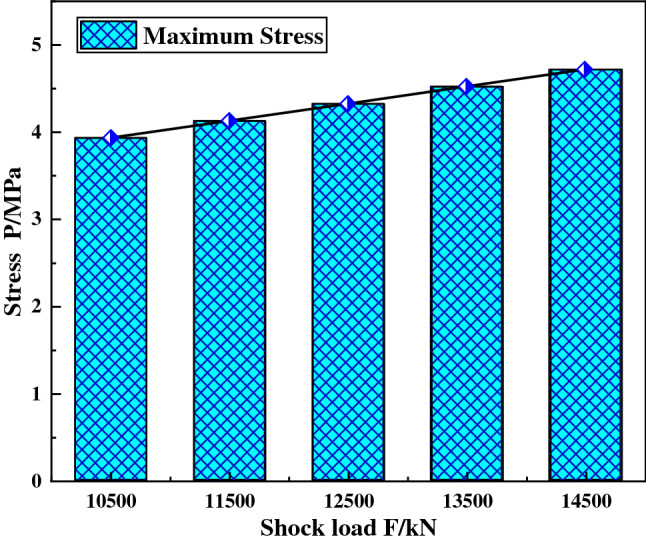
The maximum stress of the foundation node under different shock force.

When the shock angle of the ball mill material is 30°, the concrete grade is C35, the shock force are set to 10,500 kN, 11,500 kN, 12,500 kN, 13,500 kN and 14,500 kN respectively. The calculation model was submitted, and the displacement curve the maximum stress diagram of the foundational node at discharge position were obtained, and as shown in Figs. 5 and 6.

It can be seen from Fig. 5 that the change rule of the foundational node displacement at the discharge position is the same under different shock force, and the node displacement decays periodically with time, and the period is about 0.8 s. This is because at the moment when the shock force is applied, the foundation is subjected to the largest force, resulting in the maximum displacement value of the foundation node at the discharge position. After the shock force disappears, the displacement of node gradually attenuates under the action of the system damping.

In addition, the analysis data shows that with the increase of the shock force, the displacement amplitude of the foundational node at the discharge position increases. The reason of that is the shock force increases, the deformation of soil foundation and concrete increases, and finally the displacement amplitude of the foundational node increases.

### Influence of concrete

When the shock angle of the ball mill material is 30°, the shock force is 12500kN, and the concrete grades are set to C15, C25, C35, C45 and C55, respectively, the dynamic responses of the ball mill foundation under different concrete grades are obtained. Table 2 shows the parameters of different concrete grades, and Table 3 shows the first ten order natural frequencies of the ball mill system under different concrete grades. It can be seen from Table 3 that with the increase of concrete grades, the natural frequency of the ball mill system gradually increases.

**Table 2:** Parameters of different concrete grades.

Parameter	Concrete grade				
	C15	C25	C35	C45	C55
Elastic Modulus/Mpa	22,000	28,000	31,500	33,500	35,500
Ultimate strength /Mpa	15	25	35	45	55

**Table 3:** The first ten order natural frequencies of the ball mill system under different concrete grades.

Order	Natural frequency /Hz				
	C15	C25	C35	C45	C55
1	18.390	19.401	19.867	19.947	20.319
2	19.274	19.989	20.311	20.521	20.619
3	28.540	28.668	28.71	28.728	28.742
4	28.762	28.781	28.791	28.799	28.803
5	29.339	29.344	29.347	29.348	29.349
6	29.635	29.641	29.644	29.645	29.646
7	33.592	33.838	33.928	33.936	33.943
8	33.904	33.948	33.977	33.998	34.078
9	34.035	34.072	34.076	34.077	34.079
10	34.418	35.722	36.118	36.131	36.142

**Figure 7:**
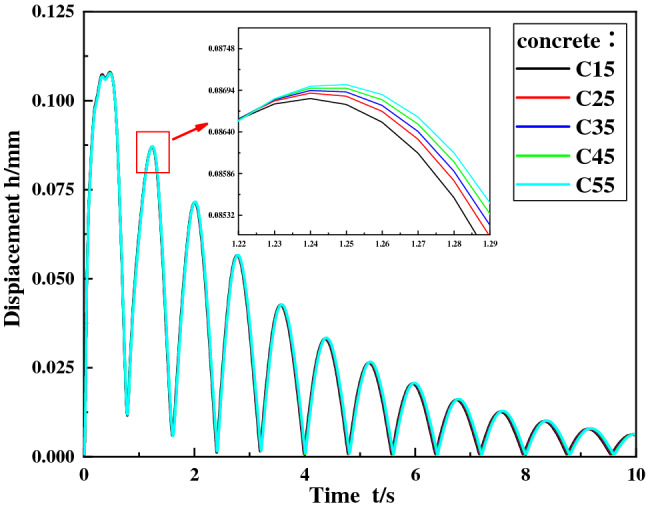
Displacement curve of foundation node under different concrete grades.

**Figure 8:**
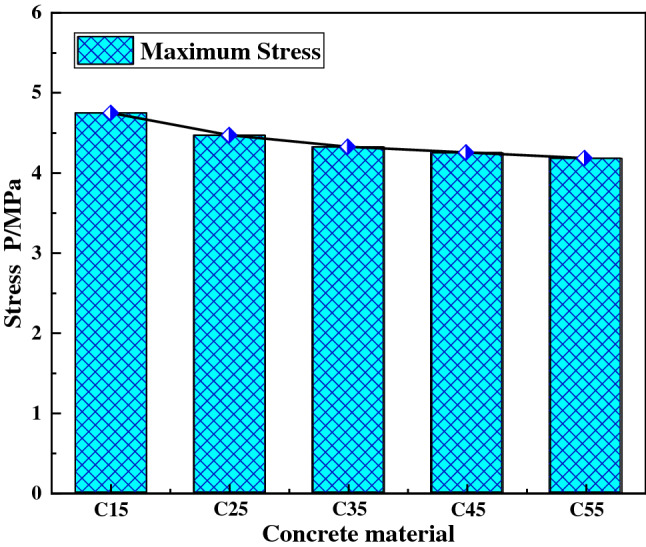
Maximum stress of foundation node under different concrete grades.

Figure 7 is the displacement curve diagram of the foundation node at the discharge position under different concrete grades, Fig. 8 is the maximum stress diagram of the foundation nodes at the discharge position under different concrete grades. It can be seen from the figures that when the concrete grade is changed from C15–C55, the displacement of the foundation node at the discharge end does not change significantly, and the maximum stress decreases slightly, indicating that the concrete grade has no significant influence on the vibration characteristics of the foundation.

### Influence of shock angle

**Figure 9:**
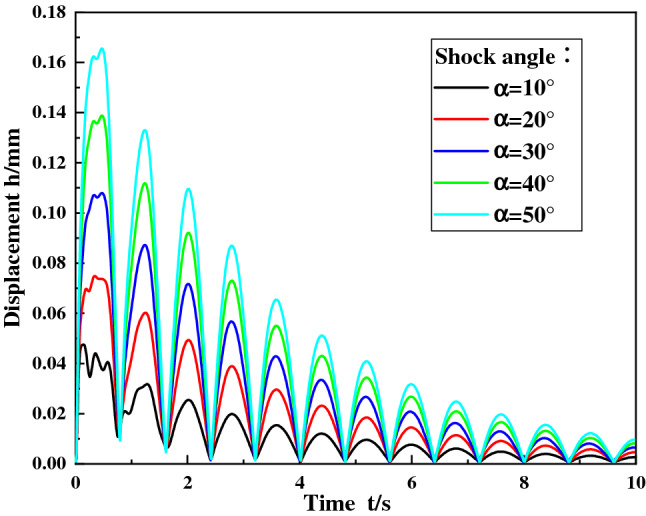
Displacement curve of the foundation node under different shock angles.

**Figure 10:**
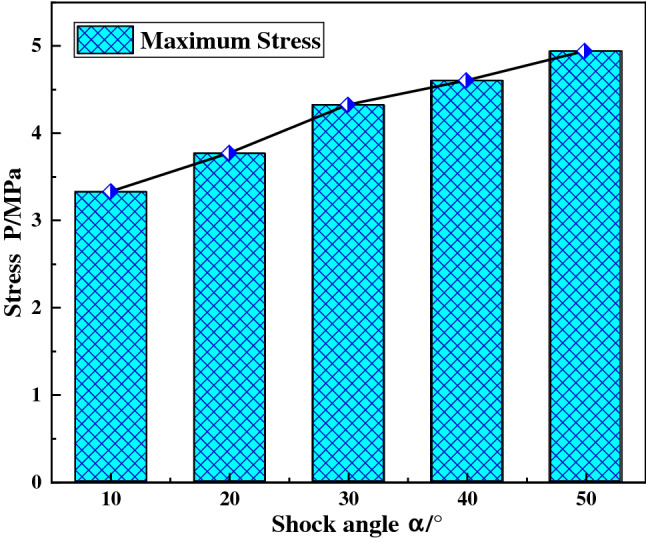
The maximum stress of the foundational node under different shock angles.

When the shock force of the ball mill material is 12,500 kN, the concrete grade is C35, the shock angles are set to 10°, 20°, 30°, 40° and 50° respectively, and the dynamic responses of the ball mill foundation under different shock angles are calculated. Figure 9 shows the displacement curve of the foundational node at the discharge position under different shock angles, and Fig. 10 shows the maximum stress of the foundational node at the discharge position.

It can be seen from Fig. 9 that when the shock angle is different, the change rule of the foundation node displacement at the discharge position is uniform, and the node displacement decays periodically with time, the period is about 0.8 s.

With the shock angle increases, the displacement amplitude of the foundational node at the discharge position increases. This is because when the shock angle increases, although the vertical force of the foundation decreases, additional bending moments will be generated, the total force on the foundation will increase, the soil foundation and concrete deformation will increase. Eventually, the displacement amplitude of the foundation node at the discharge position increases. It can be seen from Fig. 10 that with the shock angle increases, the maximum stress value of the foundation node at the discharge position increases.

### Reinforcement and verification of foundation

#### Reinforcement scheme

According to the calculation, the maximum displacement of the ball mill foundation mainly occurs at the discharge end during the transient shock, and the maximum displacement difference between the discharge position and the feed position node can reach 26 μm. It will seriously affect the operation of the ball mill and increase the damage rate of the faulty parts. In order to solve the above problems, a reconstruction scheme of the foundation is proposed.

**Figure 11:**
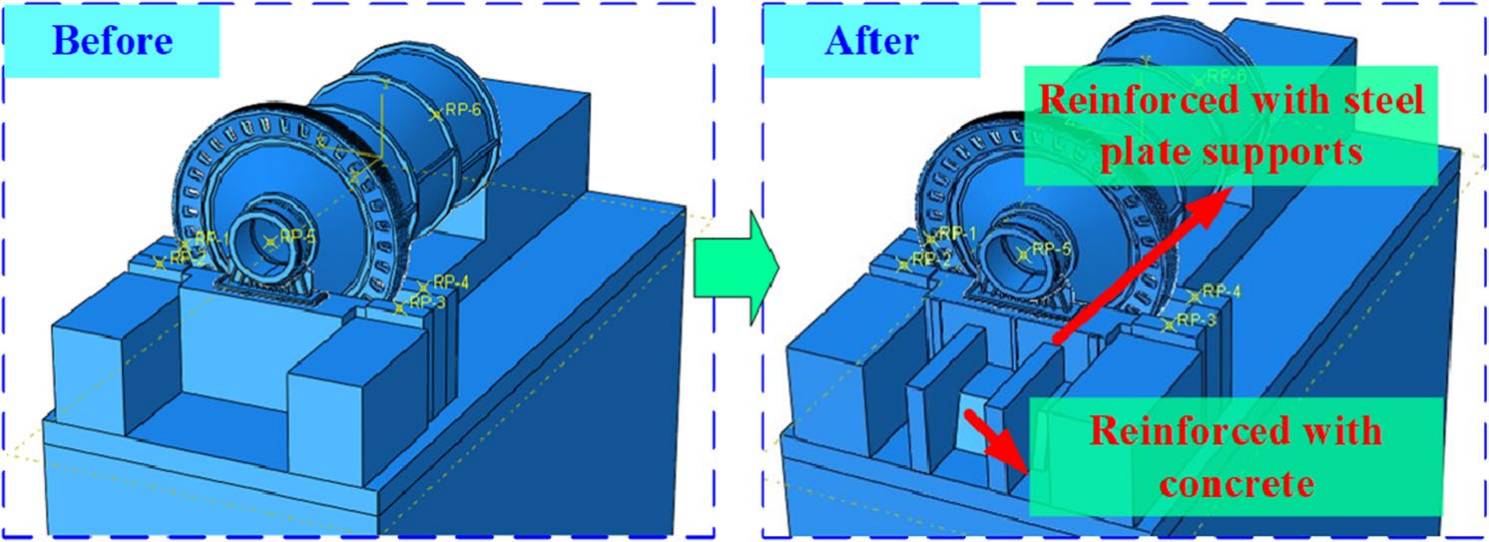
Reinforcement scheme of ball mill foundation.

The ball mill renovation plan is put forward as follows: the wall of the bearing seat at the discharge position of the ball mill is reinforced with a steel plate bracket, and the side of the wall is reinforced with a concrete block. The foundation reinforcement scheme is shown in Fig. 11.

### Comparison of results before and after improvement

**Figure 12:**
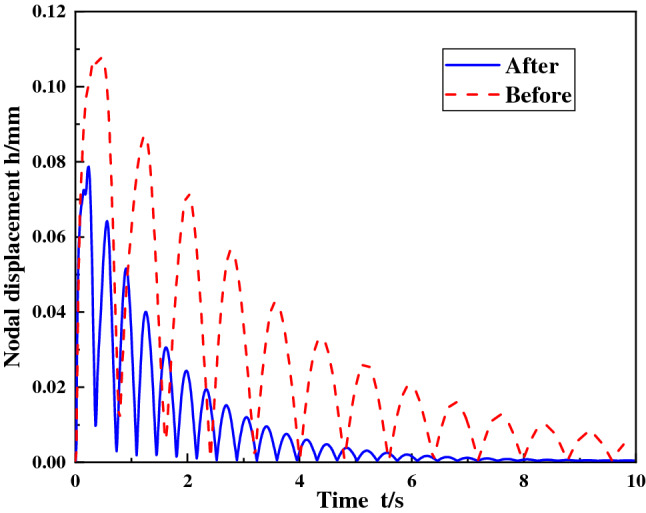
Displacement curve of the foundational node.

**Figure 13:**
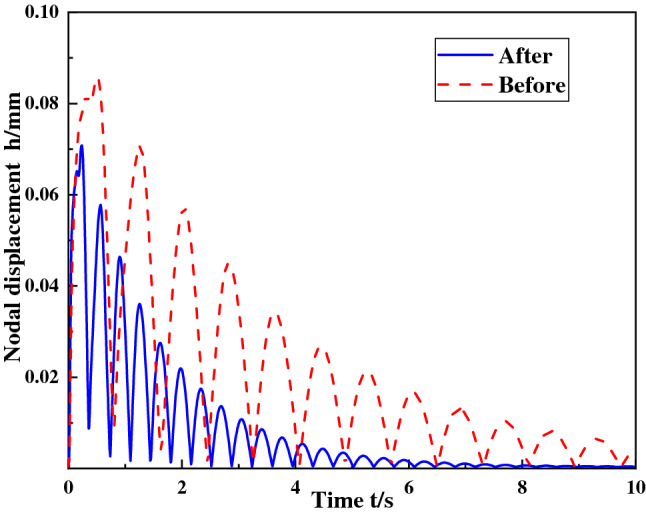
Displacement curve of the foundational node at discharge position at feed position.

After the improved foundation and other components were imported into the ABAQUS software, the shock vibration response of the ball mill foundation was calculated when the shock angle of the ball mill material was 30°, the concrete grade was C35, and the shock force was 12500 kN. Figures 12 and 13 are the displacement curves of the foundational nodes of the discharge position and the feed position before and after the improvement. It can be seen from the figure that after the foundation improvement, the node displacement of the cylinder discharge position is reduced to 78.3 mm, and the node displacement at feed position is reduced to 70.5 μm, and the improved foundation returns to the stable position earlier than before the improvement. In addition, according to the calculation, the stress of the foundational node at the discharge position after the improvement is 3.236 MPa, which is about 25% lower than the 4.325 MPa before the improvement. From the above data, it can be seen that the foundation improvement plan can greatly improve the contact characteristics between the ball mill and the foundation, and improve the stability of the foundation.

### Validation of the model

**Figure 14:**
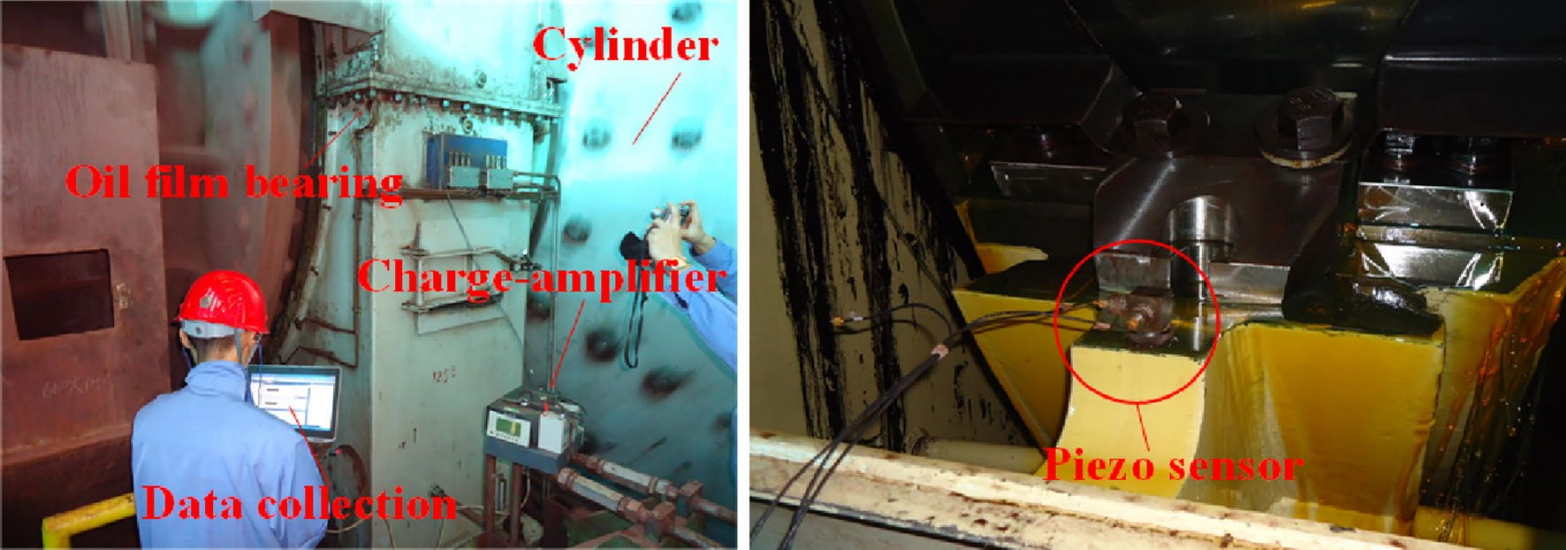
Field test diagram.

The foundational reinforcement scheme has been completed at the Yuanjia Village Iron Mine of Taiyuan Iron and Steel Co., Ltd. In order to verify the correctness of the model and scheme, relevant tests and comparative analysis of important nodes on site were carried out. In the data acquisition, the Danish B&K piezoelectric vibration sensor is used to extract the ground vibration signal, and then the signal is amplified by the Danish B&K charge amplifier, and the Belgian LMS data acquisition system is used to realize the statistics of the data. The schematic diagram of the specific instrument is shown in Fig. 14.

By measuring the four points of the ball mill foundation of φ7.32 m × 12.5 m model, the displacement data and simulation values of nodes in different orientations of the foundation when the shock force is 12500 kN and the shock angle is 30° are shown in Table 4. According to the data in the table, the error between the field test data and the finite element calculation value is small, and the simulation results are basically consistent with the field test data, so the correctness of the finite element model is verified to a certain extent. It indirectly verifies the reliability of the effect of the foundation reinforcement scheme.

**Table 4:** Simulation and test data.

Node	Before			After		
	Simulation value/μm	Experimental value/μm	Relative error	Simulation value/μm	Experimental value/μm	Relative error
Discharge position Main bearing seat bottom node	111.6	110.8	1.6%	80.5	78.6	2.4%
	109.7	107.4	2.3%	75.8	77.1	1.7%
Feeding position Main bearing seat bottom node	86.5	88.1	1.8%	69.6	107.4	2.1%
	87.4	85.6	2.1%	68.3	67.3	1.5%

## Conclusion

In this paper, a foundation dynamic model of the ball mill is established based on the finite element software, and the influence of shock force, concrete grade and shock angle on the vibration characteristics of the ball mill foundation is analyzed, and a transformation scheme is proposed. The following conclusions are drawn:

1. With the increase of the shock force, the basic displacement of the discharge position of the ball mill increases, the maximum stress increases.
2. After changing the concrete grade, the foundation displacement at the discharge position of the ball mill is unchanged, and the maximum stress is slightly reduced. The concrete grade has no significant effect on the vibration characteristics of the ball mill foundation.
3. With the increase of the shock angle, the displacement of the foundational node at the feed position of the ball mill increases, the maximum stress increases.
4. After the structural improvement of the ball mill foundation, the local displacement of the ball mill foundation is reduced, and the motion stability of the ball mill foundation is enhanced.

## Data Availability

All data generated or analyzed during this study are included in this article. All of the figures, materials, and data within the manuscript are original and owned by authors.
